# Recurrent Hematochezia Due to Ileal Varices: A Case Report

**DOI:** 10.7759/cureus.80479

**Published:** 2025-03-12

**Authors:** Sabrina Ho, John Azizian, Kai Tey

**Affiliations:** 1 Internal Medicine, University of Arizona College of Medicine, Tucson, USA; 2 Gastroenterology and Hepatology, University of Arizona College of Medicine, Tucson, USA

**Keywords:** cirrhosis, ileal varices, ir guided embolization, portal hypertension, transjugular intrahepatic portosystemic shunt (tips

## Abstract

Ectopic varices, specifically ileal varices, are a rare cause of gastrointestinal bleeding in cirrhotics. We present a case of a 42-year-old female with a history of cirrhosis complicated by esophageal varices and hepatic encephalopathy who initially presented with anemia in the setting of hematochezia. After several diagnostic methods, she was ultimately confirmed to have ileal varices, underwent placement of a transjugular intrahepatic portosystemic shunt (TIPS) and varix embolization, and her hematochezia resolved. This case highlights a rare presentation of ileal varices that were only discovered after extensive workup and required a mixed therapeutic approach via interventional radiology.

## Introduction

Gastrointestinal bleeding in cirrhotics is often associated with esophageal varices, dilated portosystemic shunts that often result due to portal hypertension. In contrast, ectopic varices (extra-esophageal varices) represent approximately 2-5% of variceal bleeding [1,2]. Ileal varices, in particular, are even more rare. Combined with jejunal varices, they make up 17% of ectopic varices [1]. Due to their location, they can be difficult to diagnose. Here, we present a rare case of ileal varices requiring a multifaceted approach in diagnosis and treatment.

## Case presentation

A 42-year-old female with past medical history significant for pancytopenia and alcoholic cirrhosis complicated by esophageal varices and hepatic encephalopathy presented with hematochezia. Over the past two months, she had been admitted at various hospitals three times for similar issues. During her first hospitalization, she underwent esophagogastroduodenoscopy (EGD) and colonoscopy. Although her colonoscopy was normal, EGD revealed esophageal varices, which were subsequently banded. A follow-up EGD four weeks later confirmed successful eradication of the varices. During her most recent admission, she was found to have severe anemia with a hemoglobin (Hgb) of 5 g/dL. Workup included a video capsule endoscopy (VCE), which identified varices in the ileum and ileocecal junction. She received four units of packed red blood cells at an outside hospital and was transferred to our facility for possible transjugular intrahepatic portosystemic shunt (TIPS) evaluation due to recurrent variceal bleeding.

**Figure 1:**
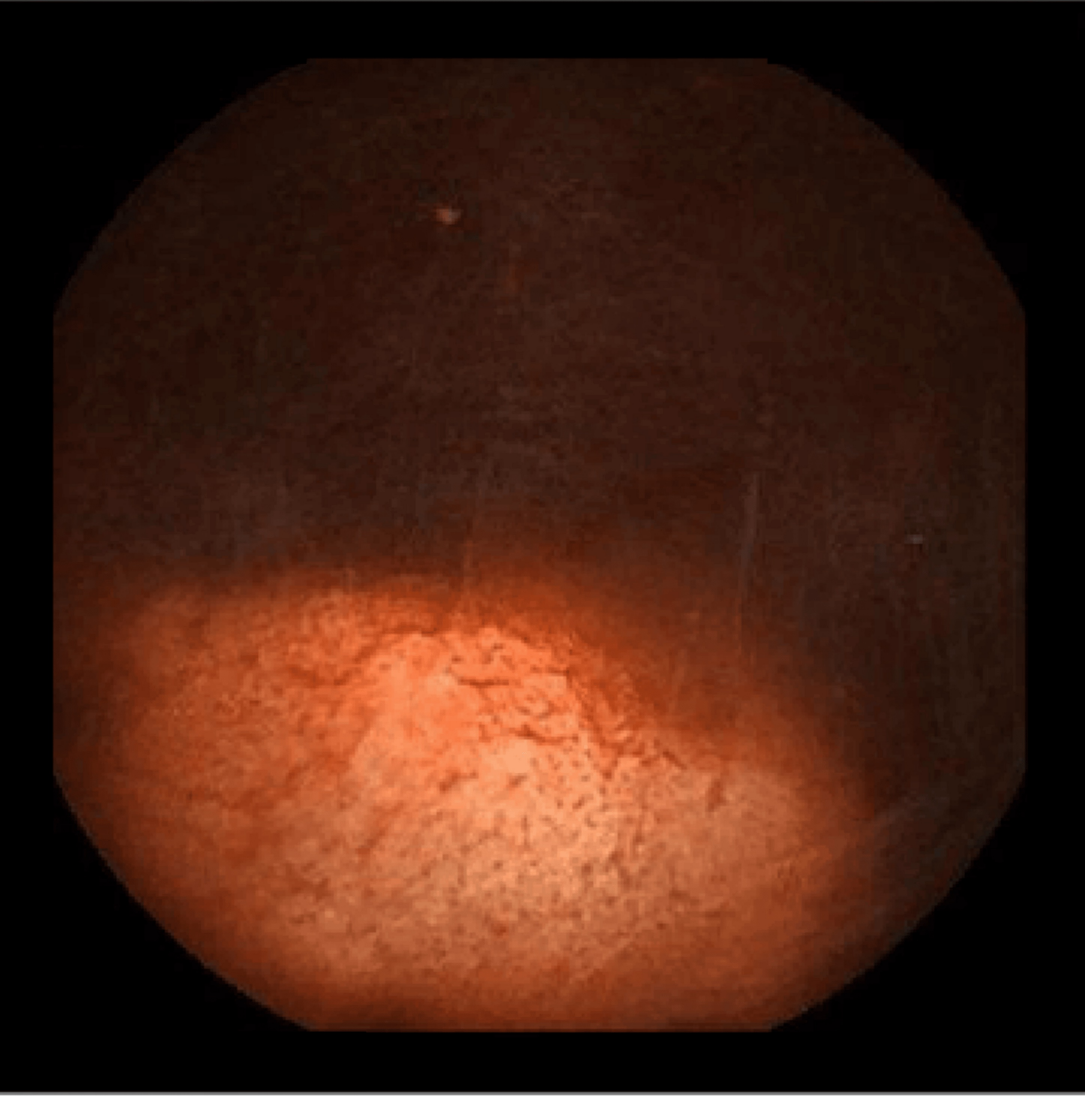
Bleeding in the ileum as seen on video capsule endoscopy.

After transfusion, the patient was hemodynamically stable with an Hgb 9 g/dL. Her Model for End-Stage Liver Disease (MELD) 3.0 score was 10. She was asymptomatic, denying any nausea, emesis, abdominal pain or further hematochezia, and her physical exam was noncontributory, prompting a repeat VCE to reassess the ileal varices seen previously. The capsule was placed. Overnight the patient had an episode of hematochezia, which continued throughout the day for a total of nine episodes. By morning, her Hgb had significantly dropped (5.7 g/dL from 7.6 g/dL); however, her vitals remained stable. Because she continued to have hematochezia, CT angiography (CTA) was performed while the VCE was read. VCE revealed two areas of bleeding: a small bleed from the stomach and a more substantial bleed in the ileum (Figure 1).

**Figure 2:**
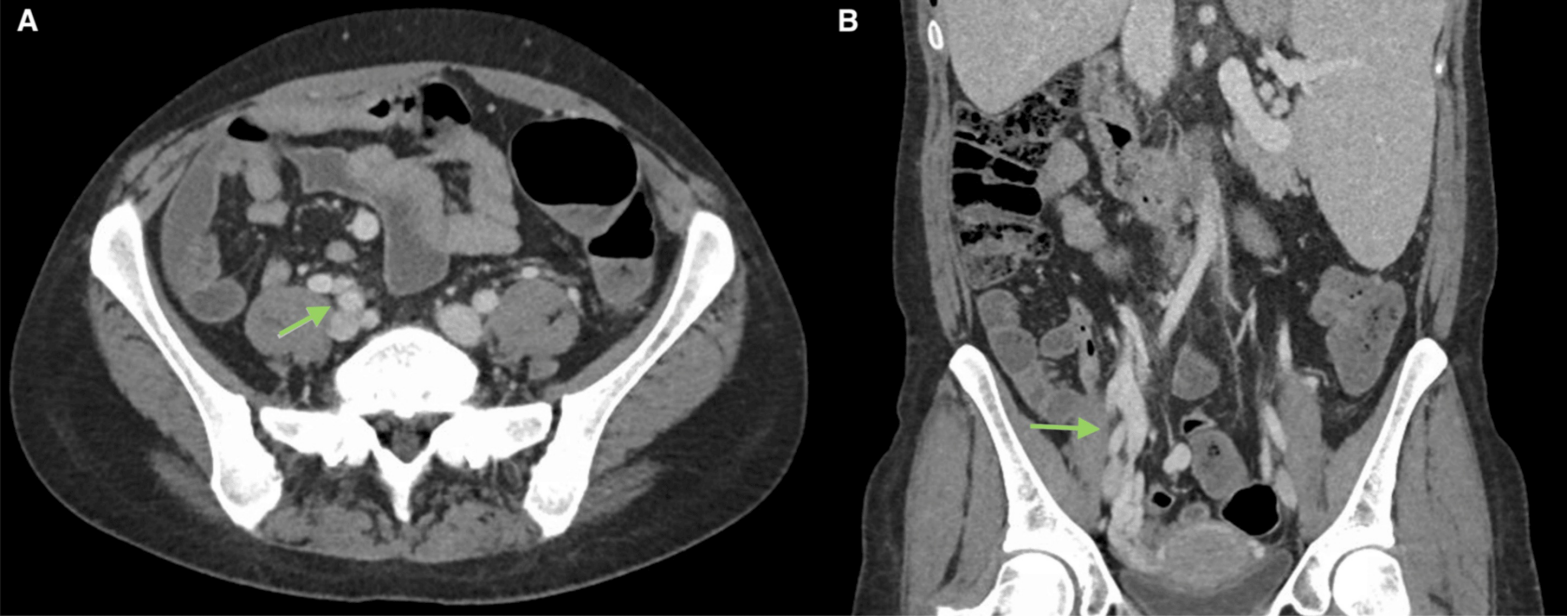
(A) Axial view of CT angiography (CTA) showing distal ileal varices. (B) Coronal view of CTA showing ileal varices and venous congestion of the uterus due to portosystemic shunting via the superior mesenteric vein to the right gonadal vein.

Due to the large amount of blood, it was difficult to visualize the source. The CTA revealed scattered areas of enhancement within both the small and large bowels, likely representing prominent vessels versus sites of possible bleeding. Additionally, large varices were observed at the distal ileal bowel loops within the right lower quadrant without evidence of active bleeding as well as significant venous congestion of the uterus due to portosystemic shunting via the superior mesenteric vein to the right gonadal vein (Figure 2).

**Figure 3:**
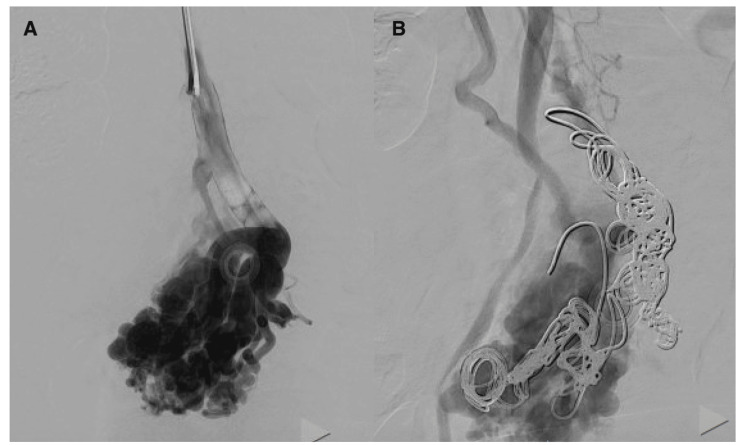
(A) Venography demonstrating ileal varix prior to embolization. (B) Venography demonstrating the ileal varix status post-embolization via 0.035-inch metallic coils with a diameter of 8 mm and Gelfoam (Pfizer Inc., New York City, NY) placement.

Interventional radiology was consulted for TIPS due to recurrent variceal bleeding with possible embolization of the ileal varix, which they performed through the right hepatic vein the next day without any complications (Figure 3).

Notably, the hepatic venous portal gradient (HVPG) was 22 mmHg pre-TIPS and reduced to 5 mmHg post-procedure. Over the next two days, the patient’s Hgb remained stable, and her hematochezia resolved. On repeat evaluation two weeks later, the patient continued to remain stable with plans to repeat imaging in a month and potentially repeat embolization if persistent varices were identified.

## Discussion

Ectopic varices refer to abnormally dilated portosystemic collateral veins in regions other than the usual gastroesophageal region [1]. Typically, they result as a complication of portal hypertension but may also develop from congenital vessel malformations, fistulas, thromboses, etc. [1]. Ectopic varices represent up to 5% of variceal bleeding episodes [3]. The most common sites include the small intestine (15% from duodenal, 17% from jejunal and ileal), colon (14%), anorectal region (8%), biliary tract, umbilicus, peritoneum (9%), or sites of previous abdominal surgery including stomas (26%) [1,3]. Previous abdominal surgery is also a significant risk factor for ileal varices, which are associated with the triad of portal hypertension, hematochezia without hematemesis and previous abdominal surgery [1,4]; however, some cases occur without any history of abdominal surgery. These varices commonly develop a shunt through the gonadal vein, less commonly through the internal iliac veins [1,5,6]. Diagnosis relies on various tools including VCE, double balloon enteroscopy, tagged red blood cell scan, and CT imaging [3]. Initially this patient was suspected to have ileal varices based on VCE at a different hospital, but the pictures on the report were inconclusive. Because the source of bleeding was identified in the ileum, we repeated VCE; however, the amount of blood obscured the view, and the diagnosis remained unclear. Ultimately, CTA confirmed the presence of ileal varices.

In terms of management, there are various possibilities: vasopressin analogues, octreotide, endoscopic intervention (sclerotherapy, thrombin injection, tissue adhesives, ligation), embolization, surgical resection, balloon-occluded retrograde transvenous obliteration (BRTO), and TIPS. In this case, a multidisciplinary evaluation was employed, and TIPS was chosen due to the patient's recurrent bleeding episodes and significant portal hypertension. Typically, a reduction in HVPG to below 12 mmHg with TIPS is known to significantly reduce the risk of bleeding from esophageal varices, but this threshold does not necessarily apply to ectopic varices [3]. Rebleeding rate has been reported to be between 23 and 25% in the first year [7], suggesting a need for additional therapy to control rebleeding. One study demonstrated that combining TIPS with embolization resulted in a lower rebleeding rate compared to using each method individually [8]. This patient had a target for embolization and therefore was able to receive both. Other case reports have also addressed ectopic varices. One study reviewed the diagnosis and management of 21 cases of ileal varices between 1982 and 2017 and found that the most common diagnostic method was through imaging [9], which was ultimately how this patient was diagnosed. The most common treatment, however, was surgical resection due to lower risk of rebleeding [9]. In this case, a multidisciplinary discussion involving hepatology and interventional radiology led to the decision to proceed with TIPS and embolization given the presence of a viable target on CTA. Ongoing monitoring with repeat imaging and potential interventions will be conducted to prevent further rebleeding.

## Conclusions

Ileal varices are a rare manifestation of portal hypertension and should be considered in the differential diagnosis of hematochezia. Diagnosis and treatment often require a multifaceted approach, involving various imaging techniques and therapeutic options as demonstrated by this case.
